# Diminishing returns as a function of the association between within-individual average performance and variance

**DOI:** 10.1016/j.heliyon.2021.e06989

**Published:** 2021-05-06

**Authors:** Kimmo Sorjonen, Guy Madison, Bo Melin

**Affiliations:** a Department of Clinical Neuroscience, Karolinska Institutet, 171 77 Stockholm, Sweden; b Department of Psychology, Umeå University, 901 87 Umeå, Sweden

**Keywords:** Ability differentiation, Average performance, Correlation, Statistical dependencies, Residual variance, Skewness, Spearman's law of diminishing returns, Variance

## Abstract

It has been demonstrated that the worst performance rule (WPR) effect can occur as a result of statistical dependencies in the data. Here, we examine whether this might also be the case for Spearman's law of diminishing returns (SLODR). Two proposed SLODR criteria are the skewness of the estimated latent ability factor and the correlation between this latent ability and within-individual residual variance. Using four publicly available datasets, covering quite different dimensions of behavior, we show that both these criteria are affected by the correlation between within-individual average performance and variance on the test scores. However, the influence of this correlation on the two criteria goes in opposite directions, which suggests that it generally might be difficult to get results that unambiguously support SLODR. These results might have far-reaching implications for the literature, to the extent that various research findings attributed to human cognitive functioning might in fact be due to trivial statistical dependencies in data. This is an important issue to address for future research.

## 1 Introduction

In the present study, we will investigate if recent findings concerning the worst performance rule (WPR) generalize to Spearman's law of diminishing returns (SLODR).

### 1.1 Diminishing returns

According to Spearman (1927) law of diminishing returns (SLODR), intelligence test scores tend to be more *g* saturated and strongly correlated among those with assumed low *g* compared to those with higher *g*. This has also been called the differentiation hypothesis (Garrett, 1946). For example, Deary et al. (1996) divided Irish schoolchildren (*N* = 10,535) into high and low scorers based on their results on the Differential Aptitude Tests (DAT) verbal reasoning subtest and calculated the amount of variance in seven other subtests (numerical ability, abstract reasoning, clerical speed and accuracy, mechanical reasoning, space relations, spelling, and language usage) accounted for by a first unrotated principal component. The amount of variance accounted for equaled 36.2% and 43.6% among the high and low scorers, respectively. Likewise, if the division was based on space relations and numerical ability, the corresponding values were 46.2% and 40.9% for high scorers and 52.9% and 46.2% for low scorers.

SLODR could be seen to suggest that intelligence is more differentiated/specialized among those with high *g*, with some individuals having, for example, especially high numeric or associative ability, while intelligence might be less specialized and more one-dimensional among those low in *g*. SLODR has received quite extensive support (e.g. Blum and Holling, 2017; Deary et al., 1996; Detterman and Daniel, 1989; Legree et al., 1996; Reynolds and Keith, 2007; Tucker-Drob, 2009), although some studies have found the opposite, that factor loadings increase with higher ability (Fogarty and Stankov, 1995; Hartmann and Teasdale, 2005).

However, most of the studies supporting SLODR have employed a method where the average correlation between test scores, or the amount of variance in test scores accounted for by a first unrotated principal component, has been compared across groups assumed to differ in average *g*. This method has been criticized for possibly giving rein to confounding factors, such as the skewness of test scores or some other difference between the subgroups that follow from how they are formed (Molenaar et al., 2010b; Murray et al., 2013). It has been proposed that SLODR is better evaluated with structural equation modeling (SEM) methods, employing the following criteria. To reflect SLODR, the data should exhibit (1) a negatively skewed latent *g*, (2) heteroscedastic subtest residuals, with a larger residual variance among those with high compared to low *g*, and (3) non-linear (quadratic) *g* loadings (Hessen and Dolan, 2009; Molenaar, Dolan and van der Maas, 2011; Molenaar et al., 2010a; Murray et al., 2013; Tucker-Drob, 2009). Research adhering to these recommendations has not reported very consistent evidence for SLODR (e.g. Molenaar et al., 2011; Murray et al., 2013).

Sorjonen and Melin (2020) demonstrate that findings in accordance with SLODR could, at least to some degree, be due to the influence of some disturbing factor, e.g. low motivation, illness, or linguistic confusion, that varies in magnitude between study participants, especially if the disturbance variable is negatively skewed. A similar threat from disturbance against the validity of the so-called intelligence-creativity threshold hypothesis has also been demonstrated (Sorjonen et al., 2019).

### 1.2 Worst performance and correlation of sorted scores rules

Following up on such extraneous causes for apparent SLODR, it is a common observation that the correlation between performance tests increases if the trials with the worst performance are used. This is known as the worst performance rule (WPR). This has mainly been documented for reaction time (RT) and its negative correlation with psychometric intelligence (see Coyle, 2003, for a review). A RT test typically consists of one to a few dozens of runs, meaning that participants get several chances to demonstrate how fast they are. It turns out that if these runs are sorted from the fastest to the slowest for each individual, the correlation with intelligence tends to strengthen from the fastest to the slowest run (Diascro and Brody, 1993; Kranzler, 1992; Larson and Alderton, 1990; Schubert, 2019; Unsworth et al., 2010; Wallert et al., 2017; but see Ratcliff et al., 2010; and Salthouse, 1998, for some non-confirming findings). This suggests that participants with low intelligence can occasionally perform very well but have an increased risk to sometimes perform extremely badly, something those with high intelligence usually manage to avoid.

The worst performance rule generalizes to the association between age and RT, where the positive correlation strengthens from the fastest to the slowest RT. In contrast, the positive correlation between intelligence or height and the participants' yearly income across several years increases from the lowest to the highest income (Sorjonen et al., 2021). As income could be characterized as a measure of performance of sorts, this finding indicates an inverse best performance rule. A trivial reason for this inversion seems to be that while the within-individual variance in RT over several runs tends to have a negative correlation with intelligence, the correlation between within-individual variance in RT and age as well as the correlation between within-individual variance in income and intelligence/height tends to be positive. These observations have led to the conclusion that the association between the correlations between sorted performances and intelligence, or some other construct, and the rank order of the performances (a so called WPR-correlation), tends to reflect the correlation between one construct and the within-individual variance of the performances on the other construct. If the latter correlation is positive or negative, so will the WPR-correlation tend to be. This phenomenon has been named the Correlation of Sorted Scores Rule (Sorjonen et al., 2020, 2021).

### 1.3 The present study

As argued above, a WPR-effect can occur merely due to statistical dependencies, in terms of a negative correlation between intelligence and within-individual variance in RT. The WPR and SLODR are similar phenomena, in the sense that they are both suggested to partly emerge due to a systematic change in the correlation between intelligence and a set of performances. The question therefore arises if also diminishing returns and ability differentiation could be provoked by similar dependencies in the data. Inasmuch as these phenomena are to some extent caused by statistical artifacts, they should manifest in any type of performance. To our knowledge, the possibility that the association between within-individual average performance and variance as an independent variable would provoke such phenomena has not been directly addressed by earlier research. The objective of the present study is therefore to assess if and how SLODR criteria are affected by such dependencies, across several different measures of performance. To assess the level of generalizability, we will include data on reaction speed, decathlon results, and earnings in addition to scores on intelligence tests, which is the standard measure of performance in research on SLODR.

## 2 Method

### 2.1 Datasets

Four publicly available datasets were used in the present study: (1) An intelligence dataset, collected from Brazilian high-school students who took 17 tests supposed to measure the broad factors (a) fluid intelligence, (b) crystalized intelligence, (c) short-term memory, (d) broad visual perception, (e) fluency, and (f) broad cognitive speed (Golino and Gomes, 2014). Data available https://dataverse.harvard.edu/dataset.xhtml?persistentId=doi:10.7910/DVN/23150); (2) A RT dataset that was collected among undergraduates at 20 U.S. and Canadian universities as well as from MTurk. Response latencies were measured when participants categorized the font color of written words that were either congruent or incongruent with the word meaning (Stroop task) (Ebersole et al., 2016). Data available https://osf.io/ct89g/). RT data are susceptible to chance events and measurement error that might produce extreme values, and response latencies that were either below the first or above the 99^th^ percentile were deleted to reduce their influence; (3) The decathlon dataset was put together by the first author from openly available results, for example at Wikipedia, from all Olympic games between 1948 (London) and 2016 (Rio de Janeiro). The dataset is available at the same location as the script (see below); and (4) an income dataset comprised data from the American National Longitudinal Survey of Youth 1979 (NLSY79), which follows individuals born between 1957 and 1964. We used data for self-reported income from 15 years between 1982 and 1999 (not quite all years included). Data available https://www.nlsinfo.org/investigator/pages/search). We decided to include only cases with complete data in the analyses, see Table 1 for sample sizes.

**Table 1:** Descriptive data, factor loadings, and model fit for the four datasets.

Statistic	Dataset			
	Intelligence	RT	Decathlon	Income
*N*^1^	225	2133	415	5299
*N* scores	17	63	10	15
Correlations				
Mean	.147	.196	.418	.597
Min	-.469	-.651	-.279	.211
Max	.635	.550	.831	.868
Loadings				
Mean	.399	.452	.703	.755
Min	.003	.173	.358	.444
Max	.633	.546	.844	.905
Model Fit				
χ^2^	285	7678	618	26544
DF	119	1890	35	90
NFI	.617	.768	.776	.699
CFI	.727	.814	.786	.700
RMSEA	.079	.038	.200	.236

1 Only including cases with complete data.

### 2.2 Data handling and analyses

**Figure 1:**
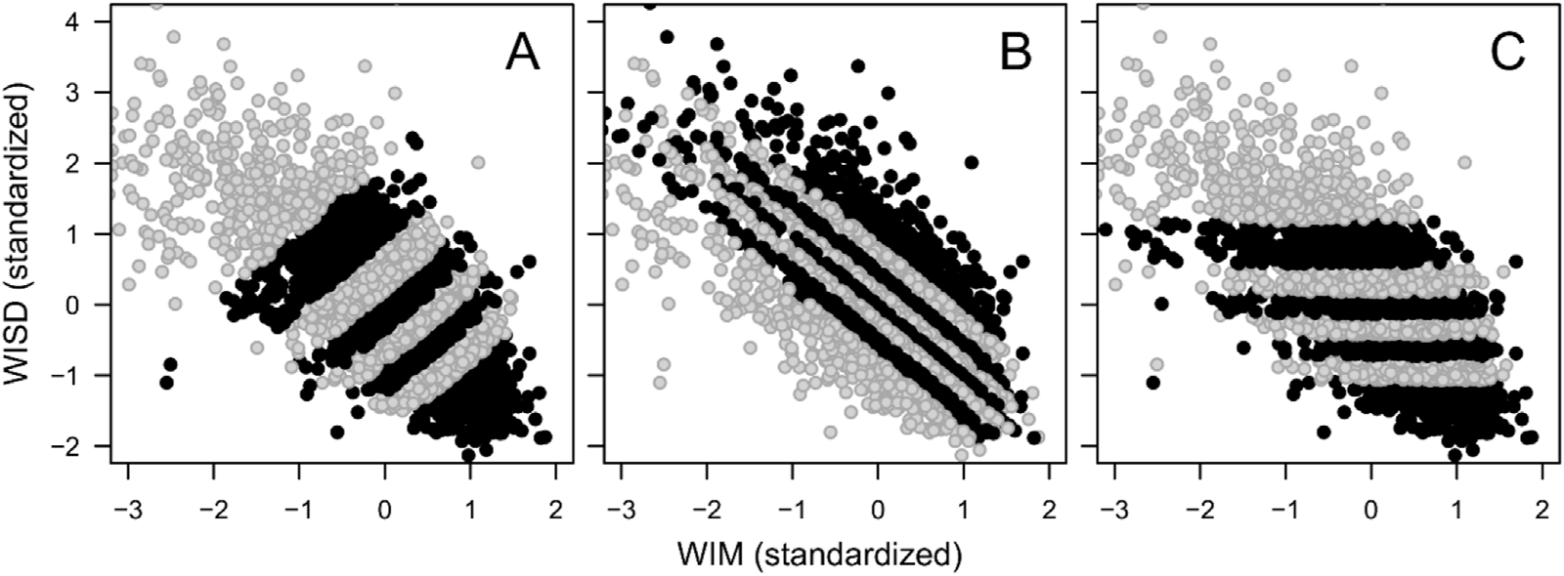
Illustration of the strategic slicing, where the RT dataset is sliced into 8 subsamples (stripes) either parallel to the antidiagonal, resulting in positive correlations between the within-individual mean (WIM) and the within-individual standard deviation (WISD) on the RTs (reversed to give measures of reaction speed) within the subsamples (A), parallel to the main diagonal, resulting in negative correlations between WIM and WISD within the subsamples (B), or parallel to the X-axis, resulting in correlations close to zero between WIM and WISD within the subsamples (C). Some extreme values are outside the axes, but were included in the analyses.

First, RTs were reversed and decathlon running times were transformed from seconds to speed (meters per second), so that higher values uniformly indicate good performance. Then, strategic slicing was applied to create subsamples parallel to the antidiagonal, main diagonal, and the X-axis (Sorjonen et al., 2021). This was expected to result in positive, negative, and essentially zero correlations, respectively, between the within-individual mean (WIM) scores and the within-individual standard deviation (WISD). The full samples were sliced into approximately equally sized subsamples ranging from 1 (i.e. the full sample) to 7 for the intelligence data, 20 for the RT data, 15 for the decathlon data, and 30 for the income data. This method of strategic slicing is illustrated with the RT dataset sliced into 3 × 8 subsamples in Figure 1.

Within the sliced subsamples, as well as the full samples, we calculated the Spearman correlation between WIM and WISD, and regressed all of the standardized scores on a single latent factor. The lavPredict function in lavaan was used to estimate the participants' scores on the latent ability factor as well as predicted values on the manifest indicators. The mean squared difference between these predicted values and observed scores were calculated for each participant and used as measures of within-individual residual variance. The main outcome variables used in the following analyses were the skewness of the latent ability scores and the correlation between the latent ability scores and the within-individual residual variances. Following Hessen and Dolan (2009), the logarithm of residual variance was used in the analyses with the correlation between latent ability scores and the within-individual residual variance as outcome. As mentioned in the introduction, quadratic factor loadings have been proposed as a third criterion of SLODR. However, they could not be used, because their inclusion rendered the analyzed models being non-convergent in most cases. We therefore considered only the other two criteria in the following. It is unfortunate that quadratic factor loadings could not be applied as a criterion in the present analyses, but we argue that an investigation of the two other criteria may still result in some interesting findings. The correlation between WIM and WISD was used as the main predictor. Data handling and analyses were conducted with R 4.0.2 software (R Core Team, 2020) employing the lavaan (Rosseel, 2012) and Hmisc (Harrell, 2020) packages. The script and the decathlon dataset are available at https://osf.io/npcfd/.

## 3 Results

Descriptive statistics for the four datasets are presented in Table 1. As the test scores in the intelligence and decathlon datasets were on different scales, all test scores in all four datasets were standardized. Although the correlations between scores tend to be positive, three of the datasets also contain variables that are negatively correlated. The factor loadings differ substantially across the data sets, and were strongest for income and weakest for intelligence. The poor fit indexes suggest that scores are affected by more than a single latent ability factor. The poor model fits are not necessarily a problem here, as the objective with the present study is to assess if SLODR criteria are systematically influenced by the correlation between WIM and WISD rather than to identify models that are good at predicting associations between performances.

**Figure 2:**
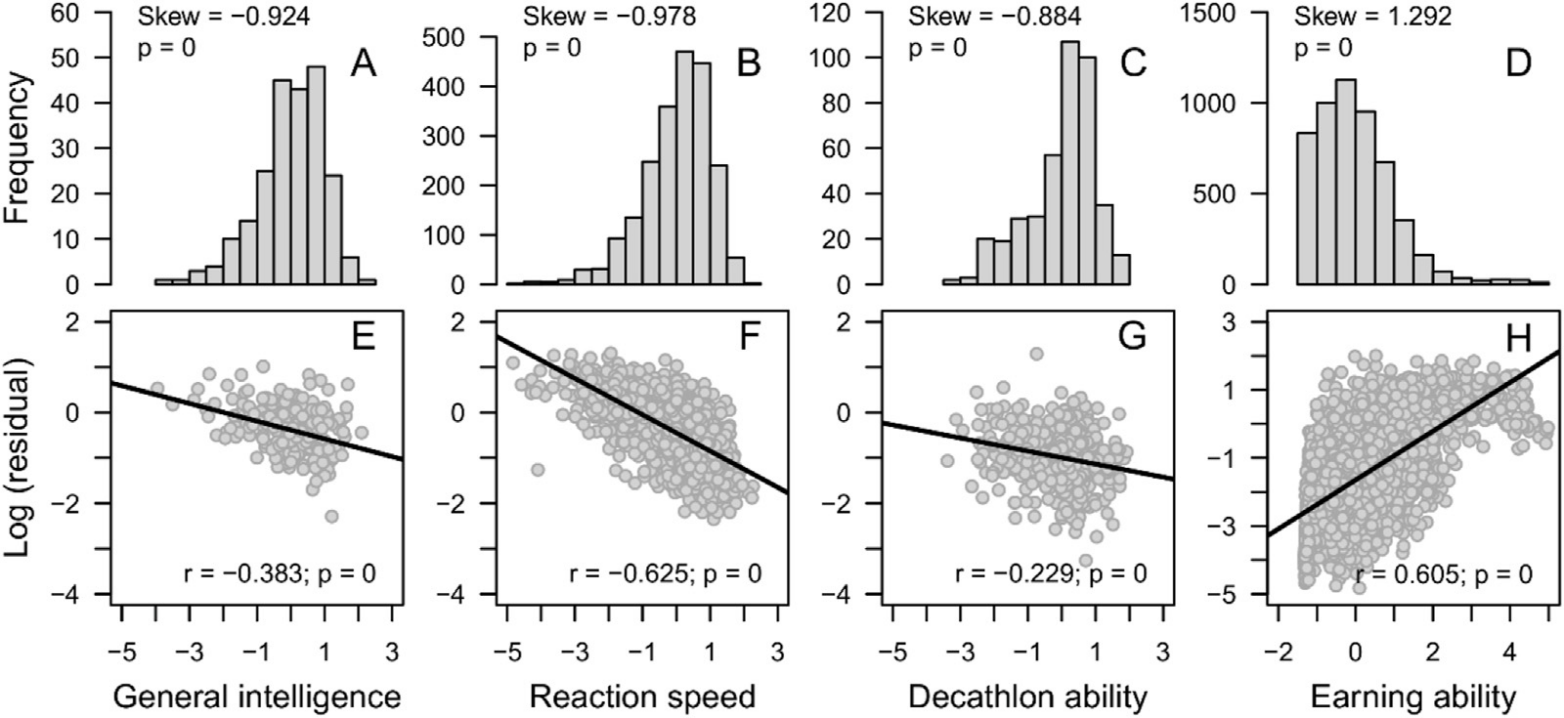
Distribution of participants' estimated scores on the latent variable (top row) and correlation between the latent variable and the logarithm of the within-individual residual variances (bottom row) in the intelligence (first column), RT (second column), decathlon (third column), and income (fourth column) datasets.

**Figure 3:**
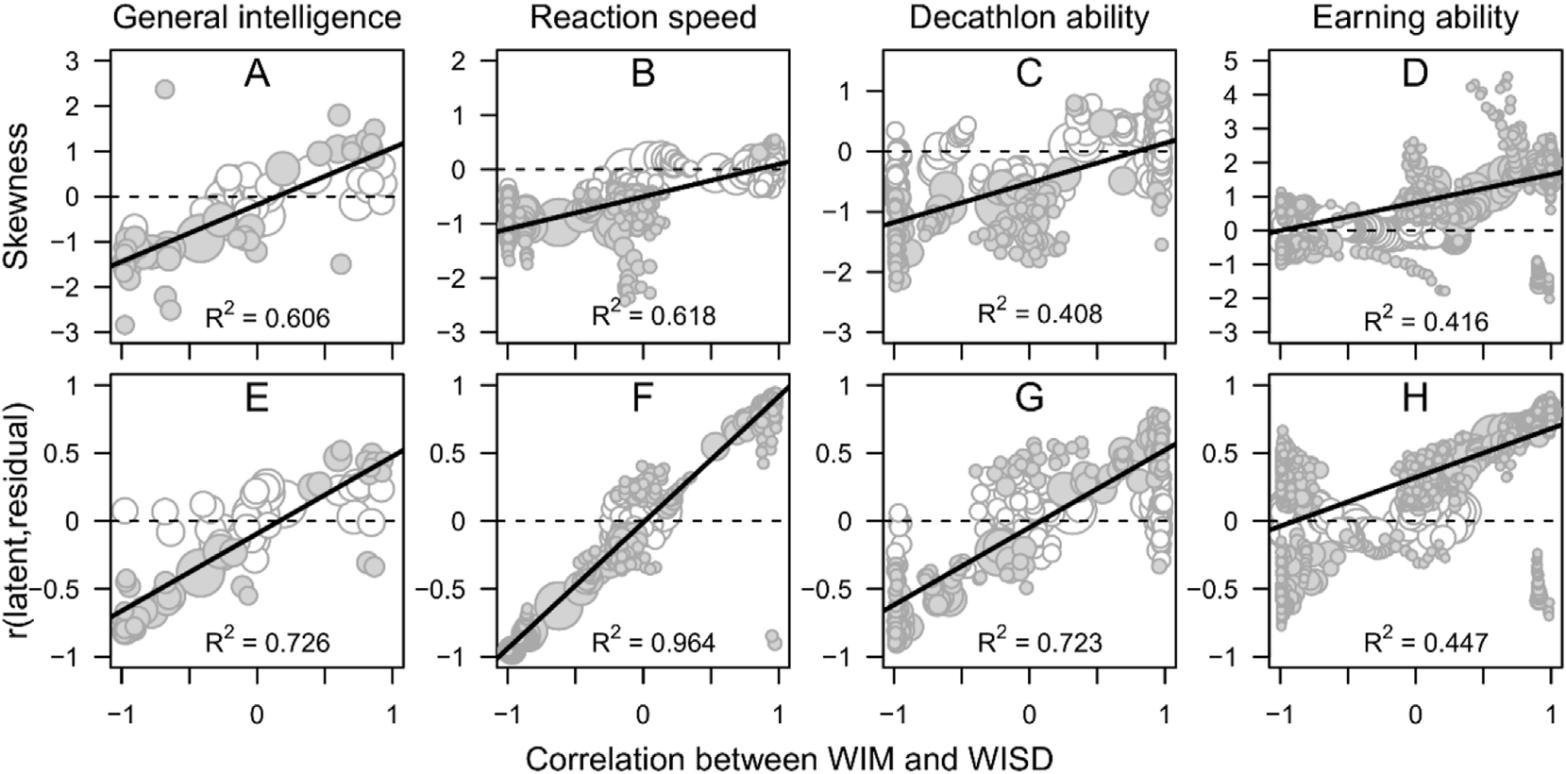
Skewness of latent ability scores (top row) and correlation between latent ability scores and the logarithm of the within-individual residual variances (bottom row) as functions of the Spearman correlation between the within-individual mean (WIM) and the within-individual standard deviation (WISD) on the items within sliced subsamples of the intelligence (first column), RT (second column), decathlon (third column), and income (fourth column) datasets. The relative size of the bubbles correspond to the size of the subsample and *N* varies between 32 and 225, between 106 and 2133, between 27 and 415, and between 176 and 5299 in the intelligence, RT, decathlon, and income datasets, respectively. White bubbles indicate non-significant (*p* > .05) and grey significant skewness/correlation.

As seen in Figure 2, the participants' latent ability scores are negatively skewed and negatively correlated with the within-individual residual variances in the intelligence, RT, and decathlon datasets. In the income dataset, on the other hand, the latent ability scores are positively skewed and positively correlated with the within-individual residual variance. This could be due to the fact that while the Spearman correlations between WIM and WISD are negative in the intelligence (*rs* = -.415, *p* < .001), RT (*rs* = -.707, *p* < .001), and decathlon (*rs* = -.215, *p* < .001) datasets, it is positive (*rs* = .600, *p* < .001) in the income dataset. This explanation was corroborated when experimentally manipulating the correlation between WIM and WISD through strategic slicing. Figure 3 shows quite clearly that both the skewness of the latent ability scores (first row) and the correlation between the participants' latent ability scores and the logarithm of their within-individual residual variance (second row) reflect the correlation between WIM and WISD within the sliced subsamples. However, both the skewness of the latent ability scores and the correlation between the participants' latent ability scores and the logarithm of their within-individual residual variance are quite reluctant to become negative in the income dataset and skewness is quite reluctant to become positive in the RT and decathlon datasets. Taken together, these observations strongly suggest that the SLODR criteria of negatively skewed latent ability scores and a positive correlation between these latent ability scores and the within-individual residual variance are functions of the correlation between within-individual average performance and variance on the test scores.

**Figure 4:**
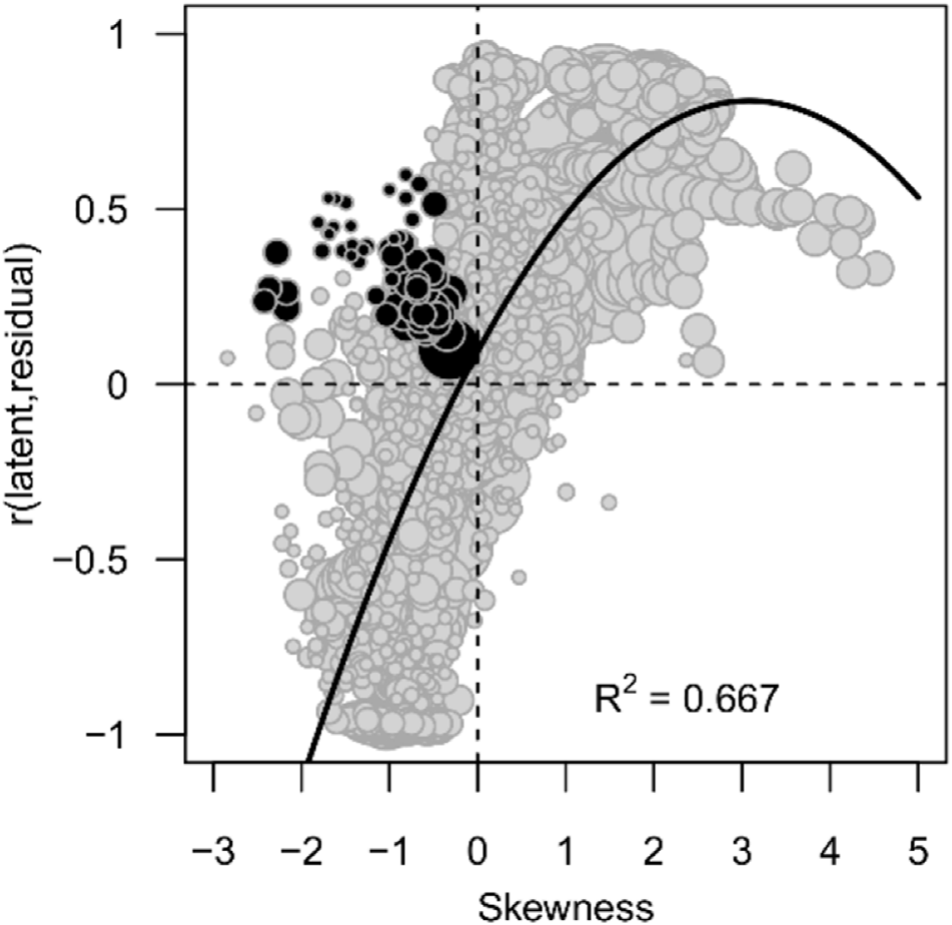
The association between the skewness of the latent ability factor and the correlation between this latent variable and within-individual residual variance, in 2469 sliced subsamples across all four datasets. The size of the bubbles corresponds to the size of the subsample. Black bubbles indicate cases for which both of the SLODR criteria of a significant negative skewness and a significant positive correlation between the latent variable and residual variances are fulfilled.

Figure 4 depicts the strong association between the skewness of the latent ability scores and the correlation between these latent ability scores and within-individual residual variance in the total 2469 sliced subsamples. The probability for both a significant negative skewness and a significant positive correlation, i.e. the SLODR criteria, weighted for subsample size, was .013 across all four datasets. The corresponding values were .008, .041, .066, and .003 in the intelligence, RT, decathlon, and income datasets, respectively.

## 4 Discussion

The objective of the present study was to assess if and how SLODR criteria are affected by the association between within-individual average performance and variance. The findings indicate that the skewness of latent ability scores and the correlation between these latent ability scores and within-individual residual variance tend to reflect the correlation between the participants' within-individual mean (WIM) and variance (WISD) on the test scores. This phenomenon seems to be general for many different types of performance, e.g. psychometric intelligence, RTs, athletic results, and income. This suggests that it might be difficult to receive results in accordance with two of the contemporary SLODR criteria, a negatively skewed latent *g* and a positive correlation between *g* and within-individual residual variance, as the likelihood for the former is increased, while the likelihood for the latter is decreased, by a negative correlation between WIM and WISD, and vice versa. This could be one of the reasons why studies employing more contemporary methods often have failed to find clear evidence of SLODR (e.g. Molenaar et al., 2011; Murray et al., 2013). Another possibility is, of course, that SLODR is generally a statistical artefact. To be clear, these are hypotheses about matters of fact, and should not be interpreted as criticism of those contemporary methods used to assess SLODR. Indeed, we have no better methods to offer. The measurement problem is further complicated by the observation that both of the SLODR criteria tend to be fulfilled if measurements are affected by negatively skewed disturbance that varies in degree between participants, as shown by simulations (Sorjonen and Melin, 2020). Specifically, this would be the case when most participants obtain scores close to their true ability while a minority obtain scores well below their true ability. Such disturbance could realistically ensue from low motivation, illness, or linguistic confusion, for example.

If tracing a possible causal chain backward, the correlation between WIM and WISD might, in turn, be a function of the skewness of the individual test scores. In the present analyses, test scores tended to be negatively skewed in the intelligence, RT (with RTs reversed to speed), and decathlon datasets, with a negative correlation between WIM and WISD. The income dataset exhibited, in contrast, a positive correlation between WIM and WISD, while the individual scores (incomes), tended to be positively skewed. This skewness might, in turn, be due to floor- and ceiling-effects. Consequently, the chain of influence would be that with ceiling (floor) effects we get negatively (positively) skewed test scores which, in turn, result in negatively (positively) skewed latent ability scores that are negatively (positively) correlated with within-individual residual variance.

Some peculiarities and limitations with the present data should be noted. The sample of Olympic decathletes is, obviously, quite exclusive, all male, and not representative of any broader population. However, our main conclusions are that the skewness of latent ability scores, as well as the correlation between these latent ability scores and within-individual residual variance, tends to reflect the correlation between within-individual mean (WIM) and variance (WISD) on the test scores. As such, it seems untenable to argue that the validity of these main conclusions is severely threatened by the exclusiveness of the decathlon sample: First, in order for Z to confound an association between A and B it needs to have an association both with A and with B. In the present case, the exclusiveness of the sample would need to have an association with the correlation between WIM and WISD, i.e. an exclusiveness of the sample × WIM (WISD) interaction effect on WISD (WIM). Furthermore, the exclusiveness of the sample would also need to have a correlation with the skewness of the latent ability scores, and with the correlation between the latent ability scores and within-individual residual variance, i.e. an exclusiveness of the sample × latent ability scores (residual variance) interaction effect on residual variance (latent ability scores). We see no compelling reasons to assume such systematic and complicated associations. Second, the main conclusion was corroborated by analyses where the correlation between WIM and WISD was experimentally manipulated through strategic slicing, and it seems quite unlikely that this experimental manipulation could have been affected by the exclusiveness of the sample. Third, the decathlon results were consistent with those based on much less exclusive samples, namely Brazilian high-school students (intelligence data), U.S. and Canadian undergraduates (RT data), and a general cohort of Americans (income data). Similar arguments could be used against potential claims that the validity of the main conclusions is compromised by the fact that (1) the intelligence dataset is quite small (and Brazilian), (2) the RT dataset was collected at multiple sites, possibly with some variation in procedures, and (3) the decathlon data is from several decades, during which professionalism in sports and standard of equipment has evolved. Furthermore, (4) some athletes contribute with data on more than one occasion in the decathlon data, (5) the worth of a dollar in the income dataset has changed over the years due to inflation, (6) scores in the datasets seemed to be affected by more than just a single latent ability factor, and (7) the individual performances may have been influenced by some disturbing factor, e.g. low motivation, illness, or linguistic confusion. Nevertheless, the slicing along the antidiagonal or the main diagonal has the same effect on the skewness of latent ability scores and the correlation between these latent ability scores and within-individual residual variance, via the correlation between WIM and WISD. That these effects persist even in the face of these differences would seem to corroborate our interpretation that the slicing and not the differences across datasets account for the found associations.

While the goals of the present study may appear narrow and the statistical methods applied are quite specific to the problem, the practical consequences might be considerable. First, the mere demonstration that this statistical artifact is likely to inflate and perhaps even cause SLODR and WPR effects call previous reports of such effects into question. Second, this prompts to examine these statistical dependencies in previously analyzed and reported datasets, aiming at quantifying their relationship to the magnitude of SLODR and WPR effects.

## 5 Conclusions

The likelihood to observe two of the proposed criteria of Spearman's law of diminishing returns (SLODR), namely a negatively skewed latent *g* and a positive correlation between this latent *g* and within-individual residual variance, are amplified by a negative and by a positive correlation between respondents' within-individual mean and within-individual variance on the test scores, respectively. This indicates that data where both of these criteria are fulfilled simultaneously might be hard to come by. This phenomenon generalizes to other measures of performance beside intelligence test scores.

The SLODR phenomenon is typically interpreted as a feature of the nature of human intelligence, where specific cognitive abilities are more independent at higher levels of general intelligence (*g*), while *g* accounts for a larger proportion of the variance across specific abilities at lower levels of *g*. The present study points to possible alternative explanations. Gauging the influence of the statistical dependencies suggested herein could possibly resolve some of the inconsistencies across studies and datasets found in the SLODR literature. This should be reasonably of interest not only for experts, but also for people in general with an interest in human intelligence and cognition.

## Declarations

### Author contribution statement

Kimmo Sorjonen: Conceived and designed the experiments; Performed the experiments; Analyzed and interpreted the data; Contributed reagents, materials, analysis tools or data; Wrote the paper.

Guy Madison, Bo Melin: Conceived and designed the experiments; Wrote the paper.

### Funding statement

This research did not receive any specific grant from funding agencies in the public, commercial, or not-for-profit sectors.

### Data availability statement

Data included in article/supplementary material/referenced in article.

### Declaration of interests statement

The authors declare no conflict of interest.

### Additional information

No additional information is available for this paper.
